# Structural and functional analysis of the finished genome of the recently isolated toxic Anabaena sp. WA102

**DOI:** 10.1186/s12864-016-2738-7

**Published:** 2016-06-13

**Authors:** Nathan M. Brown, Ryan S. Mueller, Jonathan W. Shepardson, Zachary C. Landry, Jeffrey T. Morré, Claudia S. Maier, F. Joan Hardy, Theo W. Dreher

**Affiliations:** Department of Microbiology, Oregon State University, 226 Nash Hall, Corvallis, 97331 OR USA; Department of Chemistry, Oregon State University, 153 Gilbert Hall, Corvallis, 97331 OR USA; Office of Environmental Public Health Sciences, Washington State Department of Health, Olympia, 98504 WA USA; Center for Genome Research and Biocomputing, Oregon State University, Corvallis, OR USA

**Keywords:** *Anabaena*, Anatoxin-a, Synteny, *Cyanobacteria*, Mobilome, Long-read sequencing, PacBio, Illumina, Tandem repeat, Structural variation

## Abstract

**Background:**

Very few closed genomes of the cyanobacteria that commonly produce toxic blooms in lakes and reservoirs are available, limiting our understanding of the properties of these organisms. A new anatoxin-a-producing member of the *Nostocaceae, Anabaena* sp. WA102, was isolated from a freshwater lake in Washington State, USA, in 2013 and maintained in non-axenic culture.

**Results:**

The *Anabaena* sp. WA102 5.7 Mbp genome assembly has been closed with long-read, single-molecule sequencing and separately a draft genome assembly has been produced with short-read sequencing technology. The closed and draft genome assemblies are compared, showing a correlation between long repeats in the genome and the many gaps in the short-read assembly. *Anabaena* sp. WA102 encodes anatoxin-a biosynthetic genes, as does its close relative *Anabaena* sp. AL93 (also introduced in this study). These strains are distinguished by differences in the genes for light-harvesting phycobilins, with *Anabaena* sp. AL93 possessing a phycoerythrocyanin operon. Biologically relevant structural variants in the *Anabaena* sp. WA102 genome were detected only by long-read sequencing: a tandem triplication of the *anaBCD* promoter region in the anatoxin-a synthase gene cluster (not triplicated in *Anabaena* sp. AL93) and a 5-kbp deletion variant present in two-thirds of the population. The genome has a large number of mobile elements (160). Strikingly, there was no synteny with the genome of its nearest fully assembled relative, *Anabaena* sp. 90.

**Conclusion:**

Structural and functional genome analyses indicate that *Anabaena* sp. WA102 has a flexible genome. Genome closure, which can be readily achieved with long-read sequencing, reveals large scale (e.g., gene order) and local structural features that should be considered in understanding genome evolution and function.

**Electronic supplementary material:**

The online version of this article (doi:10.1186/s12864-016-2738-7) contains supplementary material, which is available to authorized users.

## Background

*Anabaena* (some isolates are also named *Dolichospermum* [1]) are filamentous, nitrogen-fixing cyanobacteria that often form blooms in eutrophic water bodies. Traditionally, they have been studied as models of multicellular development in bacteria [2]. Their ability to fix both carbon and nitrogen makes them a key part of the biogeochemical cycle. Further, they can produce a range of bioactive secondary metabolites, which have been shown to threaten public health whenever toxic blooms occur in drinking or recreational water bodies [3, 4].

Anatoxin-a is one of the most toxic secondary metabolites produced by *Anabaena* species [5]. It acts as a nicotinic acetylcholine receptor agonist in animals, paralyzing muscles and causing death by asphyxiation [6]. The toxin is synthesized via a polyketide synthase (PKS) pathway encoded by a cluster of at least eight genes [7]. Anatoxin-a is known to be synthesized by five genera of *Cyanobacteria*: *Anabaena* (*Dolichospermum*), *Oscillatoria*, *Aphanizomenon*, *Cylindrospermum*, and *Phormidium* [8]. The entire PKS gene cluster has been sequenced and confirmed to produce anatoxin-a or a variant (homoanatoxin-a and dihydroanatoxin-a) in *Anabaena* sp. strain 37, *Oscillatoria* sp. strain PCC 6506, and *Cylindrospermum stagnale* PCC 7417 [4, 7–9]. We describe the isolation of a novel anatoxin-a-producing *Anabaena* from Anderson Lake, Washington State, USA, *Anabaena* sp. WA102.

Many cyanobacterial genomes remain in draft form (51 according to [10]). *Cyanobacteria* genomes are often resistant to standard assembly approaches when using Illumina short-insert DNA libraries, due to the fact that they have a large percentage of mobile elements (as much as 11 % of the genome) that repeat throughout the genome [11]. These repeats, and other types of repetitive DNA, are nearly identical in sequence and longer than the insert size of typical DNA sequencing libraries. This causes ambiguous alignment and scaffolding of contigs on either side of the repeat and fragments the genome assembly [12]. While most of the gene content of these genomes properly assembles, reads from mobile element regions usually do not and are omitted from analysis. Structural variation in the genome, such as large deletions or tandem duplications, is also obscured in unfinished genome assemblies. Until recently, the only methods that have spanned repeat regions and produced finished *Nostocaceae* genomes have been Sanger sequencing and hybrid assembly of 454 and Illumina sequencing libraries that require laborious extra finishing steps. Increasing access to long-read sequencing platforms will circumvent these problems and help to close complex bacterial genomes in a single assembly step [13].

We describe a PacBio sequencing dataset of 8.5 kbp average read length that was used to finish and close the genome of *Anabaena* sp. WA102. We compare the long-read sequencing results to genome assembly from short-read sequences and describe structural features of potential physiological relevance that are missed by short-read sequencing. We also compare the complete genome of the cultured isolate (Dec 2014) to the population genome of the dominant anatoxin-a-producing *Anabaena* in Anderson Lake (Jul 2012).

## Results

### The *Anabaena* sp. WA102 culture and genome

**Fig. 1:**
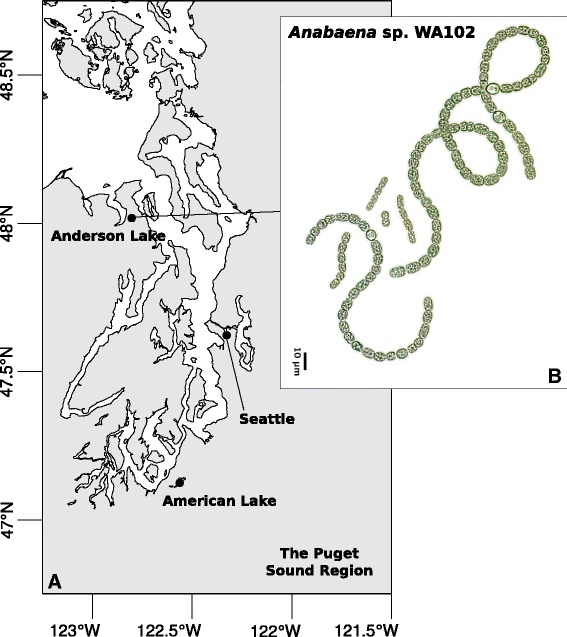
**a** A map of the Puget Sound region in Washington State, USA. *Anabaena* sp. WA102 was isolated from Anderson Lake at 48.0190 N, 237.1963 W on the Olympic Peninsula. **b** A brightfield micrograph of *Anabaena* sp. WA102 at 200x magnification. Vegetative cells measure 7.1 by 6 *μ*m on average. Colonies are heterocystous because the culture is maintained in nitrogen-free medium (BG-11_0_)

*Anabaena* sp. WA102 was isolated from a water sample collected during a cyanobacterial bloom in Anderson Lake in Jefferson County, Washington, USA on May 20th, 2013 (Fig. 1a). Anatoxin-a levels in the lake were 12.5 *μ*g/L. The non-axenic culture was first established in BG-11_0_ medium, then a single contiguous colony - assumed to be clonal - was isolated from the established culture and serially propagated in BG-11_0_. Colonies from the culture are heterocystous due to lack of nitrogen in the medium and have mean vegetative cell dimensions of 7.1 by 6 *μ*m (Fig. 1b). LC-MS/MS analysis showed that the culture produced anatoxin-a, with no detectable homoanatoxin-a nor dihydroanatoxin-a (Additional file 1: Figure S1).

**Fig. 2:**
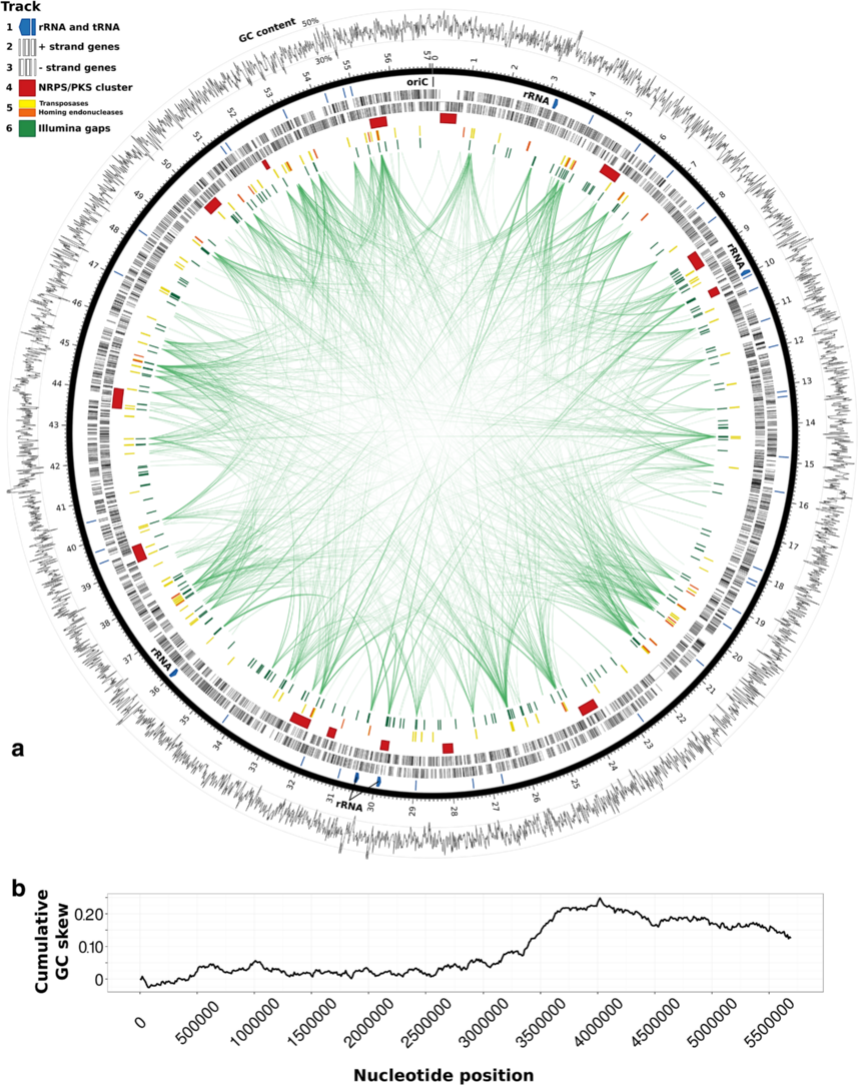
Plot of the *Anabaena* sp. WA102 genome. **a** The genome is plotted as a black ring with demarcations every 100 kbp. Average GC content in 10 kbp non-overlapping windows is plotted outside of the genome ring. The first track within the genome ring includes the location of the oriC and RNA elements. The oriC was predicted to lie downstream of *dnaA* among DnaA-binding motifs. The following two interior rings denote predicted protein-coding sequences, first on the positive strand (clockwise) and then on the negative strand (counter-clockwise). NRPS-PKS clusters identified by antiSMASH are shown as red tiles in the fourth interior track. Mobile elements - homing endonucleases and transposases - are plotted on the fifth interior track as orange and yellow tiles, respectively. Contigs from the binned Illumina genome of the culture (Fig. 6) were aligned to the closed genome and 229 gaps in the Illumina assembly are represented as green tiles in the sixth interior track. Green arcs across the center connect repeated regions in the genome, determined by blastn alignment of the finished genome against itself. Note that repeat regions often coincide with gaps in the Illumina assembly. **b** Genome-wide plot of cumulative GC skew. GC skew was averaged across 1 kbp non-overlapping windows of the genome and then cumulatively summed. Minimum and maximum points on the cumulative GC skew plot should indicate oriC and terC, respectively. However, the signal from the cumulative GC skew is weakened, preventing precise prediction of oriC, terC, and the replicon arms

DNA extracted in December 2014 (19 months after culture establishment) was used to construct a library of size-selected fragments (over 8 kbp) sequenced on four PacBio SMRT cells. A total of 1.13 Gbp with an average read length of 8.5 kbp was sequenced (Table 1 and Additional file 1: Figure S2). Two contigs representing the 5.7 Mbp chromosome and a 76.5 kbp plasmid that make up the complete *Anabaena* sp. WA102 genome were *de novo* assembled from the output of two PacBio SMRT cells (Fig. 2a). At an average nucleotide coverage of 72.0x, the average Phred quality score for the genome is 81.86 (a 1.5×10^8^ probability of an erroneous nucleotide).

**Table 1:** Summary statistics of sequencing data and binned *Anabaena* genomes

Sample	Sample	Library prep/	Seq	*Anabaena* sp. genome					
	date	Seq platform	output						
			(Gbp)						
				N50	Mean	No.	Max	Total	Unique
					cov.	contigs	contig	length	core
							(nt)	(nt)	genes
*Anabaena*	Dec	Blue							
sp. WA102	2014	Pippin/	1.13	5,715,573	50x	3*	5,705,437	5,807,452	104
culture		PacBio							
est. from	Dec	TruSeq/							
Anderson	2013	HiSeq2000	3.83	15,892	129x	819	66,878	5,698,213	105
Lake		100bp PE							
May 2013									
WA25	July	TruSeq/		Shotgun					
	2012/	HiSeq2000	30.1	metagenome					
	Anderson	100bp PE		of surface	-	-	-	-	-
	Lake			lake water					
*Anabaena*									
sp. AL93									
culture	Jan	Nextera/							
est. from	2013	MiSeq	1.36	46,264	149x	314	133,848	5,757,055	105
American		250bp PE							
Lake									
1993									

PE indicates paired-end reads. *Three contigs include the chromosome, plasmid, and the contig representing the insertion variant with the *xseA* gene

The average GC content of the *Anabaena* sp. WA102 chromosome is 38.4 %. There are 5091 predicted genes on the chromosome, including 4667 protein-coding sequences (1824 of which encode hypothetical proteins), 365 pseudogenes, 5 ribosomal RNA operons, and 43 tRNA genes (Table 1). DnaA boxes and a surrounding AT-rich region identify a single putative origin of replication from nucleotides 1457–1702. The genome has an unusual GC skew pattern (Fig. 2b) that does not allow for *terC* site prediction, as also seen with some other cyanobacteria [14, 15]. rRNA operons are scattered throughout the chromosome, not concentrated near the origin of replication, and in one case oriented against the presumed direction of replication. If *Anabaena* sp. WA102 is oligoploid like many cyanobacteria [16], then there may be less need to encode highly expressed genes such as the rRNA operons near the origin of replication to increase their copy number or orient them to optimize transcription during replication. The plasmid is 76.5 kbp long (1.3 % of genome) and has an average GC content of 37.7 %. There are 88 genes encoded on the plasmid, including 75 protein coding sequences, the majority of which are hypothetical proteins (57) or pseudogenes (13), and no rRNA or tRNA genes (Table 1).

### Comparison of *Anabaena* sp. WA102 long- and short-read genome assemblies

DNA from the *Anabaena* sp. WA102 non-axenic culture was extracted in December 2014 (7 months after culture establishment) and used to construct an Illumina TruSeq metagenome. That library was sequenced as 100nt paired-end reads on the HiSeq 2000 instrument, yielding 3.83 Gbp of total sequence, of which 738 Mbp (19 %) mapped to the closed *Anabaena* sp. WA102 PacBio genome assembly. A draft *Anabaena* sp. WA102 genome was extracted from an assembly of this short-read Illumina sequencing data using the mmgenome package. The draft genome is not complete, but the sum length of contigs in the draft genome is within 1 % of the length of the closed *Anabaena* sp. WA102 genome. When the draft genome is aligned against an HMM database of essential, universally conserved, bacterial genes from the mmgenome package, 105 essential genes found in other members of the *Nostocaceae* are also found in the new genomes (compared with 104 essential genes in the closed *Anabaena* sp. WA102 genome, see Table 2). This suggests that the draft genome is nearly complete and representative of actual gene content. Using blastn, 819 of 820 contigs in the *Anabaena* sp. WA102 draft genome align to the closed reference genome (e-value ≤10^−30^), further suggesting that the draft genome assembly has little contamination. Some of the contigs in the draft genome overlap when aligned to the closed genome, forming 230 regions of contiguous coverage with 229 gaps that are scattered around the circular genome (Fig. 2a).

**Table 2:** Summary of *Anabaena* sp. WA102 genome (Genbank:CP011456-7) annotation according to the Prokka script and NCBI Prokaryotic Annotation Pipeline

*Chromosome*				
*Category*	*Element*	*NCBI*	*Prokka*	*Manual*
Protein-coding genes:	Total	4667	5175	NA
	Hypothetical proteins	1824	2187	NA
	Transposases	79	82	130
	Homing endonucleases	7	30	30
	Histidine kinases	25	26	NA
RNA genes:	rRNA operons	5	5	NA
	tRNAs	43	44	NA
	Riboswitches	2	NA	NA
Pseudogenes:	Total	365	NA	NA
	Hypothetical proteins	186	NA	NA
	Transposases	29	NA	NA
	Homing endonucleases	6	NA	NA
	Histidine kinases	1	NA	NA
*Plasmid*				
Protein-coding genes:	Total	75	96	NA
	Hypothetical proteins	57	66	NA
	Transposases	3	2	NA
	Homing endonucleases	0	0	NA
Pseudogenes:	Total	13	NA	NA
	Hypothetical proteins	10	NA	NA
	Transposases	0	NA	NA
	Homing endonucleases	1	NA	NA

The gap regions sum to 34,166 bp (0.6 % of the reference genome), containing 97 genes. Over half of these (56 genes) have more than one copy in the genome, including 26 genes from a single cluster of transposases. Many single-copy hypothetical genes that coincide with gaps have low complexity regions. Most gaps (green tiles on Fig. 2a) coincide with long repeat regions in the genome, whose multiple copies are connected by green arcs (Fig. 2a). The repeat regions include the five rRNA operons, genes encoding transposons and homing endonucleases, and other repeat regions discussed in more detail below. In some cases gaps coincide with GC-rich regions. These results agree with previous observations of gaps in Illumina assemblies due to long repeat regions and regions of low nucleotide complexity [12]. The large number of contigs generated from the short-read Illumina sequences emphasizes the prevalence of long repetitive elements in the *Anabaena* sp. WA102 genome and the value of long-read sequencing technologies in producing finished genomes. This is further demonstrated by observations of tandem repeats in the long-read assembly, observation of structural variants in the population, analysis of genome synteny with another closely related *Anabaena* genome, and a full count of mobile elements within the genome (described below).

### The *Anabaena* sp. AL93 culture and genome

*Anabaena* sp. AL93 is an anatoxin-a producing strain isolated in non-axenic culture from a toxic bloom in American Lake, Washington in 1993 (MA Crayton, personal communication). It provides local geographical context for *Anabaena* sp. WA102, since American Lake is only 100 km from Anderson Lake. It also provides some evolutionary context as a close relative of *Anabaena* sp. WA102 (see phylogeny below). The genome was sequenced with 1.36 Gbp of Illumina MiSeq 250-bp paired-end reads. Contigs representing 5.7 Mbp of the *Anabaena* sp. AL93 draft genome were binned using the mmgenome package to yield a nearly complete genome with 105 essential genes according to the database in the mmgenome package.

### Phylogenomic relationship between *Anabaena* sp. WA102, AL93, and other fully sequenced *Nostocaceae*

**Fig. 3:**
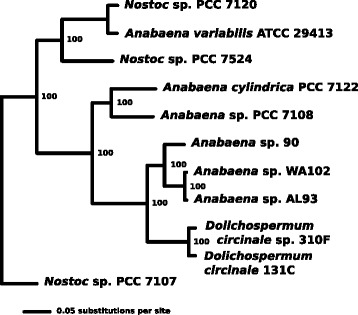
A phylogenomic tree constructed from amino-acid alignments of single-copy orthologs present in all genomes of some of the fully sequenced members of the *Nostocaceae*

The closed genome from *Anabaena* sp. WA102 and the draft genome from *Anabaena* sp. AL93 can be placed phylogenetically among draft and full genomes from members of the *Nostocaceae*. The ancestral relationship of eleven genomes from the *Nostocaceae* was hypothesized with a phylogenomic tree based on 1408 clusters of unique orthologs from each genome (Fig. 3). Unanimity among 1000 tree constructions yielded 100 % bootstrap support for every internal node. The tree was rooted at *Nostoc* sp. PCC 7107, according to [10]. *Anabaena* sp. WA102 and *Anabaena* sp. AL93 are most closely related to each other. They form a distinct clade with *Anabaena* sp. 90, a microcystin toxin-producing strain from Finland [15].

### Comparing gene content and metabolic capabilities of *Anabaena* sp. WA102 and AL93 with other *Nostocaceae* genomes

**Fig. 4:**
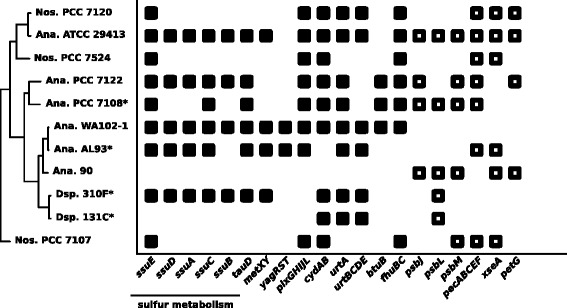
KEGG orthologs (KO) differentially represented among the compared *Nostocaceae* genomes. All proteins from each *Nostocaceae* genome were mapped to the online KO database. Orthologs with significant differences among the genomes were highlighted in the above table for comparison. *Nostocaceae* genomes are arranged according to the phylogenomic tree for easy comparison. The *Anabaena* sp. WA102 genome encodes a sulfur metabolism cluster absent or incomplete in 6 out of 11 *Nostocaceae* genomes

The gene contents of *Anabaena* sp. WA102 and closely related *Nostocaceae* genomes were also assigned to metabolic pathways using the KEGG ortholog database. All genes necessary for nitrogen fixation (*nifDKH*) were found throughout these genomes. Figure 4 highlights metabolic pathways with differential representation in *Anabaena* sp. WA102 and its relatives. Differences in sulfur metabolism are evident among the genomes. The *ssu* operon, which is involved in transport and metabolism of organic sulfur compounds [17], was intact in *Anabaena* sp. WA102. It was absent or incomplete in 6 of 11 *Nostocaceae*, including *Anabaena* sp. 90. *ssuABCDE* and *tauD* (taurine metabolism) are in the same gene cluster in *Anabaena* sp. WA102 and are likely co-regulated. *Anabaena* sp. WA102 also possesses the *fhuBC* genes, which encode two parts of the ferric hydroxamate ABC transporter. The presence of these genes suggest that *Anabaena* sp. WA102 is well equipped to import organic sulfur compounds and iron from the environment. This may provide a competitive advantage in providing the iron-sulfur clusters that are necessary for nitrogen fixation in niches with low sulfate availability.

Other genes present in *Anabaena* sp. WA102 but not in *Anabaena* sp. 90 or other *Nostocaceae* (Fig. 4) may also provide competitive advantage under certain conditions. *btuB* is necessary for vitamin B12 uptake from the environment [18]. The *urtABCDE* cluster allows uptake and metabolism of nitrogen-rich urea [19]. *cydAB* encode the cytochrome *bd*-type oxidase, which has been shown to be necessary for *Nostoc* sp. PCC 7120 survival under nitrogen-limited conditions and is hypothesized to scavenge oxygen in heterocysts to prevent oxidation of nitrogenase [20]. The presence of *pixGHIJL* genes, which encode a phototactic system, suggests that *Anabaena* sp. WA102 is positively phototactic and likely motile [21]. In support of this hypothesis, *Anabaena* sp. WA102 encodes a twitching-motility pilus gene *pilT*, and a pilus assembly gene *pilC* (loci AA650_16975 and 16980). Gas vesicle genes present in two clusters (loci AA650_0781 to 07850 and AA650_07865 to 07870) support mobility through buoyancy control [22].

A number of metabolic genes are absent from *Anabaena* sp. WA102, but present in *Anabaena* sp. 90 or other *Nostocaceae*. *pecABCEF*, the genes responsible for phycoerythrocyanin synthesis [23]), are absent from *Anabaena* sp. WA102 but present in its close relative *Anabaena* sp. AL93 [24]. Phycoerythrocyanin is a photosynthetic pigment that absorbs light maximally at 575nm (green light) and confers a competitive advantage in coastal and freshwater environments where phytoplankton and turbid waters absorb much of the red light that is maximally absorbed by the ubiquitous phycocyanin pigment [23]. These two strains can be distinguished by their pigments, a critical element in niche adaptation. Both strains encode genes to synthesize the phycobilins phycocyanin and allophycocyanin, but only *Anabaena* sp. AL93 encodes the genes for phycoerythrocyanin synthesis. The absence of a phycoerythrocyanin operon suggests that *Anabaena* sp. WA102 would not compete well in shade from other photosynthetic organisms or deeper and murkier water because it cannot efficiently absorb green light. Rather, it may avoid shade or deeper water by positive phototaxis to the lake surface driven by gas vesicle buoyancy. The *psbJLM* components of the photosystem II apparatus are intermittently distributed throughout the *Nostocaceae* in this study but are completely absent from *Anabaena* sp. WA102. Different combinations of light harvesting genes in each genome, without a phylogenetic pattern, suggest that they are selected for under different light conditions and perhaps horizontally transferred.

### Capacity for synthesis of anatoxin-a and other secondary metabolites

**Fig. 5:**
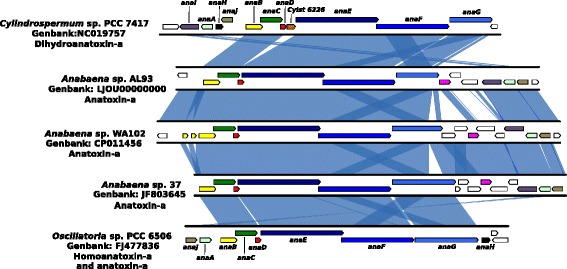
Nucleotide alignment of anatoxin-a clusters from *Cyanobacteria*. *anaA-G* and *anaI* are all conserved in *Anabaena* sp. WA102 and *Anabaena* sp. AL93, though *anaH* is missing from both. The 5’ region of *anaB* and upstream promoter region is triplicated in *Anabaena* sp. WA102. The anatoxin-a cluster from *Anabaena* sp. WA102 is most similar to that from *Anabaena* sp. 37. The three *Anabaena* strains share a gene of unknown function downstream of *anaG* (colored pink). The *anaG* genes differ in size, correlated with different variants of anatoxin-a. Shorter variants of AnaG omit or truncate a putative methyl transferase domain. The *anaF* and *anaG* genes share a region of 86 % nucleotide identity that is likely a homologous protein domain. *Anabaena* sp. WA102 and AL93 encode two of the shortest *anaG* genes and produce anatoxin-a, *Cylindrospermum* sp. PCC 7417 produces dihydroanatoxin-a (likely due to the unique gene Cylst 6226), and *Oscillatoria* sp. PCC 6506 primarily produces homoanatoxin-a

Cyanobacteria produce many secondary metabolites, including products of nonribosomal peptide synthetase (NRPS) and polyketide synthase (PKS) genes. Much concern about freshwater cyanobacterial blooms stems from their production of toxic secondary metabolites. Fourteen gene clusters in the *Anabaena* sp. WA102 genome encode putative secondary metabolite synthesis proteins (Fig. 2a and Additional file 1: Table S1). Anatoxin-a is made by proteins encoded in cluster eleven located between nucleotides 4,362,415 and 4,392,159, confirming that *Anabaena* sp. WA102 indeed is able to produce anatoxin-a, as detected by LC-MS/MS (Additional file 1: Figure S1). The *anaA-G* genes in this 30 kbp cluster are syntenous with homologs in *Anabaena* sp. 37 and *Anabaena* sp. AL93 (Fig. 5). However, genes *anaA*, *anaI*, and *anaJ* are rearranged between the *Anabaena* anatoxin-a clusters and the *Oscillatoria* and *Cylindrospermum* clusters [7].

Comparing *ana* clusters between *Anabaena* sp. WA102, *Anabaena* sp. AL93, *Anabaena* sp. 37, *Cylindrospermum stagnale* sp. PCC 7417, and *Oscillatoria* sp. PCC 6506 showed differences in the *anaG* gene (Fig. 5 and Additional file 1: Figure S3). The AnaG protein plays a key role in determining the anatoxin variant produced [7]. AnaG adds an acetyl group and either one or two methyl groups to the bicyclic thioester precursor, forming either anatoxin-a or homoanatoxin-a, respectively. *Oscillatoria* sp. PCC 6506, which produces 99 % homoanatoxin-a and 1 % anatoxin-a, possesses the largest methyltransferase domain in AnaG. The smaller AnaG methyltransferase domain in *Anabaena* sp. 37, a producer of anatoxin-a [8], is evidently not involved in homoanatoxin-a synthesis. The AnaG methyltransferase domain is missing entirely in *Anabaena* sp. WA102 and *Anabaena* sp. AL93, which are also producers of anatoxin-a (Fig. 5 and Additional file 1: Figure S3). In *Cylindrospermum* sp. PCC 7417, which produces dihydroanatoxin-a, AnaG lacks the methyltransferase domain as well as the phosphopantetheine transferase domain on the extreme C-terminus (Additional file 1: Figure S3). In the same strain, an oxidoreductase gene, *Cylst6226*, not present in the other *ana* clusters, is present (Fig. 5) and implicated in dihydroanatoxin-a synthesis [7]. Note that annotation of genes *anaH-J* differs between [7] and [4]; we have chosen to follow [7].

The anatoxin-a synthetase gene cluster from *Anabaena* sp. AL93 revealed an organization most similar to that of *Anabaena* sp. WA102, although the AL93 AnaG gene is shorter in the C-terminal region. There are also differences in genes situated between *anaG* and *anaI*, which include genes not thought to be involved in anatoxin synthesis. Notably, all clusters (not shown for *Oscillatoria* sp. PCC 6506 in Fig. 5, but referred to in [7]), share a MATE efflux pump homolog (*anaI*). MATE efflux pumps encoded within the saxitoxin gene cluster are known to export saxitoxin, another toxic secondary metabolite, from the producing cell [25]. They may play a similar role with anatoxin-a.

### Lack of synteny with *Anabaena* sp. 90

Among the completely sequenced *Anabaena* genomes, *Anabaena* sp. WA102 is most closely related to *Anabaena* sp. 90, sharing an average nucleotide identity (ANI) of 91.5 % and 2331 gene homologs. Despite this relatively close relationship, there are major differences in overall genome architecture. Whereas the *Anabaena* sp. 90 genome has two chromosomes of 4.33 and 0.82 Mbp, *Anabaena* sp. WA102 has a single chromosome. Local nucleotide alignment showed that there is little long-range synteny between the two *Anabaena* genomes (Additional file 1: Figure S4).

**Fig. 6:**
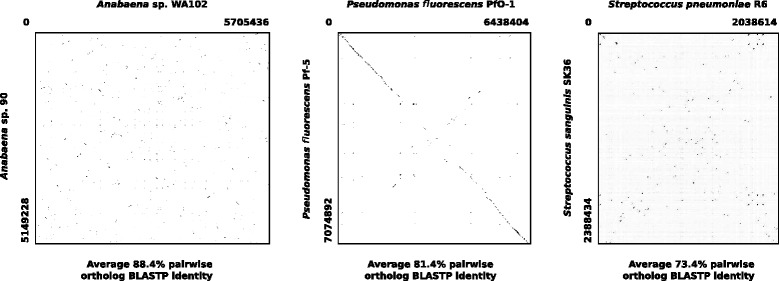
Dotplots and average ortholog similarity for pairwise comparisons within three bacterial genera. Dotplots illustrate preservation or absence of long-range nucleotide similarity (synteny) between paired genomes from *Anabaena* in this study and *Pseudomonas* and *Streptococcus* (originally compared in Novichkov et al., 2009)

Novichkov et al. illustrated common paradigms of synteny between genomes within a genus using dotplots [26]. Aligning genomes between species of *Pseudomonas* yielded long stretches of synteny, but aligning genomes between species of *Streptococcus* showed no synteny. Those dotplots are recreated and shown beside the dotplot for *Anabaena* sp. 90 and WA102 (Fig. 6). Orthologs from each pair of aligned genomes were aligned by BLASTP, showing that average amino acid identity between orthologs of the *Anabaena* genomes was the highest (Fig. 6). The dotplot of the *Anabaena* genomes is very fragmented, although these genomes are relatively closely related. The distinct X-shape to dotplots of *Pseudomonas* and *Streptococcus* genomes indicate chromosomal inversions around the origin of replication [27]. This pattern is missing in the dotplot of *Anabaena* genomes, indicating the infrequency or absence of these inversions. Figure 6 indicates that the *Anabaena* genomes have experienced a relatively faster rate of recombination versus point mutation. This is not uncommon among bacterial genomes but varies among different taxa [28]. Length distributions of the local colinear blocks (LCB’s) from alignments calculated by Mauve (Additional file 1: Figure S5) support the general disruption of gene order between *Anabaena* sp. WA102 and 90. The largest local colinear blocks (Additional file 1: Table S2) encompass biosynthetic gene clusters and a cryptic prophage discussed below. The LCBs are not clearly bounded by either repeat sequences or mobile elements, which does not lend a clear explanation for their rearrangement between the two bacteria.

In addition to long-range shuffling, we also detected local rearrangement of genes within clusters. For instance, an LCB at nucleotides 1,992,912-2,007,469 that includes thirteen genes in *Anabaena* sp. WA102 corresponds to the region between nucleotides 3,575,881-3,591,878 in *Anabaena* sp. 90 that includes fourteen genes (Additional file 1: Figure S6). Genes in this syntenous region are putatively involved in complex carbohydrate biosynthesis and export (being mostly glycosyltransferases and including an ABC transporter). Of these, two glycosyltransferases, an acyltransferase, and a hypothetical protein are unique to *Anabaena* sp. WA102 and six glycosyltransferases are unique to *Anabaena* sp. 90. The remaining nine genes in *Anabaena* sp. WA102 and eight genes in *Anabaena* sp. 90 are homologous or share homologous domains. Two transposases are responsible for interrupting just one portion of synteny in this region, leaving 4 breaks in synteny unexplained. This suggests that recombination interrupts synteny even in otherwise conserved gene clusters, though the mechanism for recombination is not always clear.

### The mobilome

**Fig. 7:**
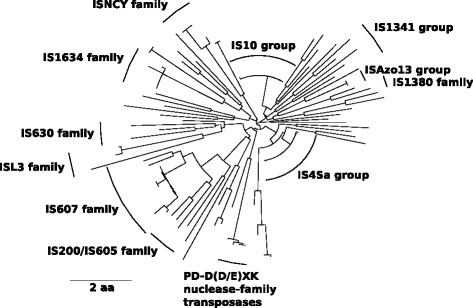
Phylogenetic tree of transposase protein sequences encoded in the *Anabaena* sp. WA102 genome. The phylogenetic relationship between 130 annotated transposase protein sequences is sketched out in the tree. Two large clades of closely related transposases dominate the tree. The IS4Sa clade includes 25 transposases and the IS10 clade includes 20 transposases, which both belong to the larger IS4 transposase family. These transposases have a DDE-type active site that facilitates cut-and-paste transposition. The IS4Sa clade has an identical terminal direct repeat sequence: CCGCCTTGTCACCCGTTAAG. The IS10 clade has the terminal direct repeat sequence: ATTCAACAYTTCTG

One hundred eight transposases (79 intact and 29 pseudogenes) were automatically annotated by the NCBI pipeline, constituting 2 % of the genome. Manual annotation with the aid of the IS Finder database [29] increased the number of intact and fragmented transposases to 130. In addition to transposases, 30 HNH homing endonuclease reverse transcriptases are encoded in the *Anabaena* sp. WA102 genome, bringing the total number of intact and degenerate mobile elements to 160. Phylogenetic relationships between insertion sequences show that two groups of closely related IS4-family insertion sequences predominate (20 in the IS10-like group and 25 in the IS4Sa-like group) among a wider representation of IS families (Fig. 7). Aligning nucleotide sequences adjacent to each side of the coding sequence of these insertion sequences revealed the unique inverted repeat sequence for each group: ATTCAACAYTTCTG for the IS10-like group, and CCGCCTTGTCACCCGTTAAG for the IS4Sa-like group. These two groups of transposases catalyze their transposition via three acidic residues in their active site: two aspartates and a glutamate, and transpose in a cut-and-paste fashion (non-replicative) [30].

Other common mobile elements found in bacterial genomes are prophages, cryptic prophages, and phage-like elements such as gene transfer agents (GTAs). No signature phage regions were detected with the PHAST phage-detection webserver. The IslandViewer 3 webserver, which detects genomic islands, highlighted an 18 kbp region between nucleotides 1,179,961 and 1,198,734. This region is also contained in the largest local colinear block (LCB) calculated by Mauve between *Anabaena* sp. WA102 and *Anabaena* sp. 90 (Additional file 1: Table S2). Within the LCB, there is a 19 kbp insertion in *Anabaena* sp. WA102 relative to *Anabaena* sp. 90. The LCB boundaries likely denote the exact boundaries of a cryptic prophage: nucleotides 1,179,211-1,198,554. This region contains a putative phage terminase large subunit that was automatically annotated by Prokka and confirmed with 100 % confidence by Phyre2 structure-guided annotation. The terminase large subunit is a component of a DNA packaging protein unique to *Caudovirales*. Within this region also lie 21 hypothetical proteins, one IS-4 family transposase, one pseudogene, and one integrase. The large proportion of hypothetical proteins is consistent with a phage origin. The integrase lies 134 nucleotides downstream of a methionine tRNA, which may have served as an integration site (attB) of the prophage. The GC content in the region is 32.9 %, lower than the genome average of 37.7 % and consistent with a horizontally transferred region that has a distinct nucleotide composition. The small size of the region, lack of other identifiable phage proteins such as capsid or tail structure proteins, and the insertion of a transposon common to the bacterial genome suggest that this region is a partly degraded cryptic prophage. Several other phage integrases were automatically annotated, but these integrases are often functionally mislabeled. Alternatively, they may be site-specific integrases native to or co-opted by the bacterial genome for functions other than prophage integration and excision. These alternative functions are likely, considering the absence of other readily identifiable phage genes near these integrases.

Besides transposons and phage-like elements, a single plasmid was identified, rounding out the mobile element complement of *Anabaena* sp. WA102. The plasmid was identified as a 92 kbp contig assembled from PacBio reads. Fifteen kbp of nucleotide sequence from each end of the contig aligned with 99 % similarity (overlapped with lower quality sequence at the extremities) and was excised from the final plasmid. The trimmed plasmid sequence is 77 kbp long, with a 37.2 % average GC content. The 88 genes on the plasmid include 75 intact and 13 pseudogenes. A *parAB* operon on the plasmid suggests that it is a low-copy plasmid (confirmed by an average read coverage less than that of the chromosome) with a well described partitioning mechanism [31]. The *parAB* operon and surrounding nucleotide sequence bears at least 86 % similarity to the *parAB* operon and its surrounding sequence on the chromosome (Additional file 1: Figure S7). Interestingly, the plasmid carries at least part of a non-ribosomal peptide synthase (NRPS) cluster. One protein within the cluster shows significant similarity to AdpD from the anabaenopeptilide cluster in *Anabaena* sp. 90 (BLASTP e-value =6.9×10^−121^). The other three biosynthetic proteins in the cluster show similarity to a malonyl CoA-acyl carrier protein transacylase, a *β*-ketoacyl synthase, and a short-chain dehydrogenase. Plasmid-borne NRPS clusters are not uncommon. A recent comprehensive survey of NRPS and polyketide synthase (PKS) clusters in all bacterial genomic data deposited at the National Center for Biotechnology Information (NCBI) revealed that 10 % of NRPS/PKS clusters in *Cyanobacteria* are located on plasmids [32]. Importantly, the plasmid encodes four putative site-specific integrases, which may facilitate integration into a bacterial chromosome. Coupled with nucleotide similarity between the plasmid and the chromosome, where site-specific integrases can also be found, this indicates that the region of plasmid similarity on the chromosome may be considered a genomic island.

### Relationship between the *Anabaena* sp. WA102 genome and the Anderson Lake metagenome

To relate the *Anabaena* sp. WA102 genome to a bloom in Anderson Lake, the WA25 metagenome was sampled from Anderson Lake on July 7th, 2012 (unpublished data). The sample was taken near the peak of a cyanobacterial bloom, when the anatoxin-a level was 187 *μ*g/L (https://www.nwtoxicalgae.org/Data.aspx). The metagenome contains a genome from a strain of *Anabaena* sp. WA102 that is nearly identical to the culture and is likely an ancestor from 10 months before the culture strain was isolated and 2.5 years before it was sequenced. Reads from the July 2012 metagenome short-read Illumina), the December 2013 culture (short-read Illumina), and the December 2014 culture (long-read PacBio) were mapped to the closed reference genome to track changes in the genome over time.

### A recent deletion event in the *Anabaena* sp. WA102 genome

**Fig. 8:**
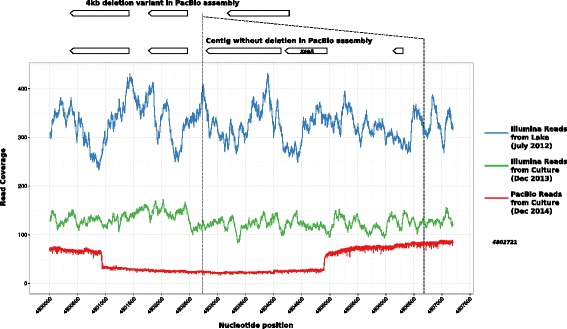
A deletion mutation was detected in the PacBio long-read assembly of the *Anabaena* sp. WA102 culture. Mapping reads to the indel region showed that the deletion occurred between nucleotides 4,800,950 and 4,804,900. The deletion arose after December 2013 and expanded through the population to roughly two-thirds of the culture population by December 2014

The length of the PacBio reads not only allowed us to close the *Anabaena* sp. WA102 genome but also revealed structural variation in the population. The 21 kbp segment between nucleotides 4,790,517 and 4,812,024 was also present (99 % similarity) on a 25 kbp contig in the PacBio assembly, reflecting the existence of a 4kbp indel variant within the genomes of the *Anabaena* sp. WA 102 culture population (Fig. 8). Mapping reads from the *Anabaena* sp. WA102 PacBio dataset showed that the contig had an average coverage of 25x, approximately one-third of the average coverage of the chromosome (73x), and that the deletion actually lies between nucleotides 4,800,950 and 4,804,900. This suggests that the deletion is present in two-thirds of the *Anabaena* sp. WA102 culture population. The indel appears to be a deletion that arose after December 2013, since the longer sequence is predominant in sequencing reads from both the July 2012 metagenome and the December 2013 culture (Fig. 8). An XseA homolog (the large subunit of exonuclease VII) and two hypothetical gene products are deleted in the variant. In well characterized *Escherichia coli xseA* mutants, there is an increased recombination phenotype [33], suggesting the same may be true for two-thirds of the *Anabaena* sp. WA102 culture population.

### Tandem repeat of the anatoxin-a *anaBCD* promoter region

**Fig. 9:**
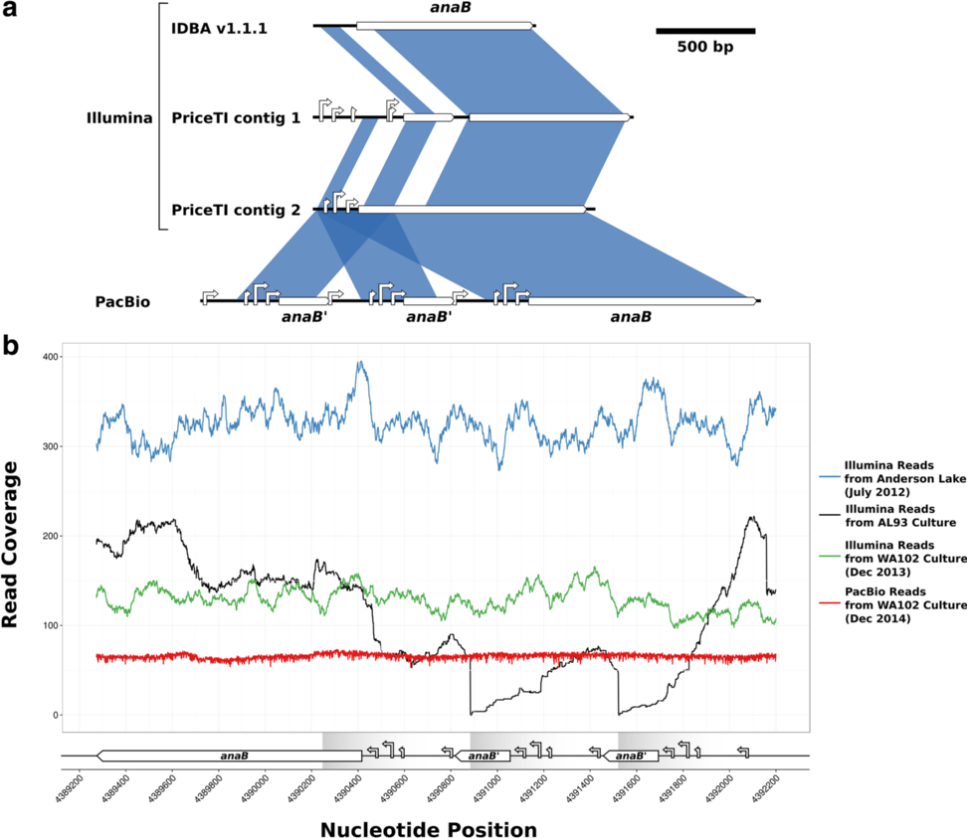
Tandem triplication of the putative anaBCD promoter region. **a** Alignment of the *anaB* gene and upstream promoter region between different assemblies of the *Anabaena* sp. WA102 culture. Promoters were identified with the Virtual Footprint online server, and only promoters with PWM alignment scores greater than 12 were plotted. The 5’ end of the *anaB* gene and upstream promoter region are triplicated in the PacBio assembly. None of the Illumina assemblies correctly assemble the tandem triplication. Assembly of 100 bp reads by IDBA v1.1.1 failed to correctly assemble the *anaB* gene and the promoter region. Assembly by PriceTI v1.0.1, using the IDBA contig to seed the assembly, produced two alternate versions of the *anaB* region. In the first version, the *anaB* gene and the upstream promoter region are both improperly assembled. In the second, the *anaB* gene and the most proximal portion of the promoter region are correctly assembled, but triplication is not assembled. **b** Read coverage across the promoter region upstream of the *anaB* gene. Illumina metagenome reads from a toxic bloom in Anderson Lake (WA25, *blue line*), *Anabaena* sp. AL93 culture (green line), and *Anabaena* sp. WA102 culture are mapped across *anaB* and its upstream promoter region. Coverage is summed at each nucleotide and illustrates the absence of two junctions formed between the triplications where the green line drops to zero for the *Anabaena* sp. AL93 culture. In contrast, both the *Anabaena* sp. WA102 culture and the Anderson Lake metagenome contain the junctions formed by the triplication because read coverage does not fall to zero at those loci. Presence of the triplication in the Anderson Lake metagenome indicates that it formed in the *Anabaena* sp. WA102 genome nearly a year prior to establishing the culture. It has been under selection in the environment and continues to be selected for in culture. *Read coverage values for the July 2012 Anderson Lake metagenome have been divided by 10 to facilitate comparison along the ordinate

Intriguingly, the anatoxin-a synthase region in the PacBio assembly of *Anabaena* sp. WA102 showed that the first 173 bp of the *anaB* gene and 398 bp upstream of the gene had been triplicated (Figs. 5 and 9). This is in contrast with the genome of *Anabaena* sp. AL93, which does not have a triplication of the *anaB* promoter region. The 398 nucleotides upstream of *anaB* include four high-scoring putative transcriptional regulation binding sites and promoters, identified *in silico* using Virtual Footprint and the PRODORIC database of position weight matrices for bacterial transcriptional regulation binding sites and promoters [34]. Assembling Illumina reads from the *Anabaena* sp. WA102 culture with IDBA v1.1.1 and PriceTI fails to correctly resolve the tandemly triplicated promoter region (Fig. 9a). To determine when this triplication arose, reads from the July 2012, Dec 2013, and Dec 2014 sequencing runs were mapped to the triplicated region (Fig. 9b). Illumina reads from the Anderson Lake metagenome and the *Anabaena* sp. WA102 culture mapped across the two unique junctions formed by the triple tandem repeats, confirming its presence as early as 2012 in Anderson Lake and also in the culture sequenced in December of 2013. In contrast, none of the reads from the *Anabaena* sp. AL93 culture mapped across the unique junctions formed by the tandem repeats (indicated by arrows in Fig. 9b). This triplication is unique to *Anabaena* sp. WA102 among all known anatoxin-a cluster sequences and has been stable for at least 2.5 years, in both Anderson Lake and under culture conditions. Toxin production has been measured in the culture (Additional file 1: Figure S1), so the tandem repeat is not interrupting transcription of the *anaBCD* operon. Instead, triplication of the putative promoter region may increase transcription of the operon.

## Discussion and conclusions

### The recently cultured toxic isolate, *Anabaena* sp. WA102, closely reflects the parent population in Anderson Lake

*Anabaena* sp. WA102 is a novel anatoxin-a-producing member of the *Nostocaceae* isolated from Anderson Lake on the Olympic Peninsula in Washington in 2013. It is in stable non-axenic culture. The *Anabaena* sp. WA102 genome is unique among sequenced *Anabaena* genomes because it was sequenced within seven months of isolation. Other *Anabaena* strains have been in culture for several decades prior to whole genome sequencing and changes in a strain’s genome can accumulate over such long periods. Sequencing a strain soon after isolation increases the relevance of the sequenced genome to the environment from which it was isolated and provides a reference point for later studies of the strain’s genome.

*Anabaena* sp. WA102 produces anatoxin-a in culture (Additional file 1: Figure S1). The toxin is produced by NRPS and PKS enzymes encoded by the *anaA-J* gene cluster. A triple tandem repeat of the *anaB* putative promoter region in the cultured isolate (Fig. 9a) is present in a nearly identical strain in the environment (July 2012 Anderson Lake sample, WA25 in Table 2), which suggests that it originates from and is relevant to the lake environment. Tandem repeats of genes and promoters commonly arise in bacterial genomes but are unstable and can collapse through homologous recombination or strand slippage at high frequency, unless the repeat is under selection [35, 36]. Thus, tandem repeats have been hypothesized to act as a crude selection-regulated response to environmental change [37–39]. Additionally, tandem repeats provide redundancy that drives the innovation, amplification, divergence (IAD) cycle that generates genetic novelty [40]. Tandemly repeated promoters, in particular, allow for promoter regions to generate or acquire new regulatory binding sites that can change the expression pattern of an operon [41]. Further study of this tandem repeat may be fruitful for several reasons. Most noteworthy is that these tandem repeats are 617nt long and identical, which makes them highly susceptible to homologous recombination that can either expand or collapse the repeats [42]. Tandem repeats tend to be deleted rather than expanded unless deletion is selected against. This instability may be exacerbated by the deletion of the *xseA* gene in part of the population (Fig. 8a), which causes a hyper recombination phenotype in *Escherichia coli*. That the tandem triplication can be detected in *Anabaena* sp. WA102 over a span of two years, including in Anderson Lake, suggests that a selective pressure in the lake and in the culture may be maintaining the triplication. Key questions are whether the tandem repeat increases expression of the *anaBCD* operon and production of anatoxin-a, and whether elevated expression is under selection. Determining the selective pressure preserving the tandem repeat in the *Anabaena* sp. WA102 culture may illuminate the function of anatoxin-a in the environment.

### Closing the genome reveals details about genome architecture

Long-read sequencing technology will increasingly allow for bacterial genomes to be assembled in a single step [43]. Closing the *Anabaena* sp. WA102 genome with as few as two PacBio SMRT cells demonstrates that it is pragmatic to use non-axenic environmental enrichments of targeted bacterial species in order to obtain their finished genomes. The long-read library (PacBio C6-P4 technology) used in this study yielded an average read length of 8.5 kbp, which is long enough to span long-repeat regions in most bacterial genomes including refractory genomes such as those of the bloom-forming cyanobacteria *Anabaena* and *Microcystis* [13, 44]. Greater access to long-read sequencing raises expectations for the quality of bacterial genome assembly and will yield new insight into the mobilome and structural variation in bacterial populations. The mobilome in many bacteria may be under-represented because mobile elements that are repeated throughout bacterial genomes cannot be assembled correctly with short-insert DNA libraries. Observing structural variation such as erosion of synteny (Fig. 6, Additional file 1: Figure S4) and accumulation of local repeats (Fig. 9) will enhance our understanding of bacterial evolution. In fact, short-insert libraries can be incorrectly assembled to suggest features that do not exist. An example of that is the missrepresentation of the *anaB* tandem repeat region in the *Anabaena* sp. WA102 genome (Fig. 9a). *De novo* assembly of short-insert genomic libraries is not sufficient to determine the number of replicons in a genome or overall gene order. Further, this method is liable to miss structural variants within a population, such as the fractional presence of an *xseA*-bearing insertion (Fig. 8a). While short-read sequencing possesses distinct shortcomings in describing structural features of a genome, nearly all single-copy genes that make up the majority of a bacterial genome can be assembled from short-read Illumina sequencing runs (Table 1 and Fig. 2a).

### Predicted ecologic profile of *Anabaena* sp. WA102

Mapping proteins from *Anabaena* sp. WA102 to the KEGG ortholog database indicates a metabolism acclimated to a nutrient-rich freshwater environment with ample sunlight. The inability to produce phycoerythrocyanin, produced by some related *Anabaena*, coupled with positive phototaxis and gas vesicle operons suggest that it competes for light by outmaneuvering other photosynthetic organisms and rising to the surface of the water to avoid niches with less green light. Competition experiments between other nitrogen-fixing autotrophs and *Anabaena* sp. WA102 could test these hypotheses. Freshwater cyanobacteria are known to secrete hydroxamate-based siderophores to chelate iron in water [45]. These siderophores, including those encoded by the *fhu* genes in *Anabaena* sp. WA102, are then transported across the cell membrane by ferric-hydroxamate transporters [46]. Efficiently scavenging sulfur and iron would help maintain iron-sulfur clusters that are heavily used in nitrogen fixation and photosynthesis, so the predicted ability of *Anabaena* sp. WA102 to assimilate organic sulfur and oxidized iron from the lake environment may confer a growth advantage in some conditions over cyanobacteria lacking *ssu*, *tau* and *fhu* genes (Fig. 4).

### Evolution of the *Anabaena* sp. WA102 genome

A genomic island and a complementary plasmid carrying novel genetic cargo (Additional file 1: Figure S7), tandem triplication of a promoter (Fig. 9), observed deletion of a 4kb fragment of the genome (Fig. 8), the ubiquity of mobile elements (Fig. 2), and the nearly total absence of synteny with *Anabaena* sp. 90 (Fig. 6) suggest that the genome is in rapid flux. The potential for the genome to radically rearrange may allow *Anabaena* sp. WA102 to respond to gradual changes in the environment, such as climate change, if such changes offer the opportunity to adjust gene expression profiles. The increased availability of closed genomes as long-read sequencing becomes more widely used will allow us to quantify the rate of recombination in genomes in *Anabaena* and in other bacteria. It will then be possible to test hypotheses for the most prevalent mechanisms and drivers of genome remodeling.

More genomes from closely related species need to be finished with long-read sequencing. These genomes can then be arranged in an alignable tight genome cluster and assayed for gene family growth and loss, and for rearrangements [47]. Alternatively, resequencing metagenomes of the original environment of *Anabaena* sp. WA102 - Anderson Lake - at regular intervals is currently feasible. This approach would generate a regular time series record of differences in the population genome of *Anabaena* sp. WA102 in its native environment with nucleotide resolution.

## Methods

### Sample collection

500 mL samples were collected from Anderson Lake, Washington State (48.0190 N, 237.1963 W) by the Jefferson County Public Health Department during the 2012 and 2013 cyanobacterial toxic bloom seasons. Samples were collected at a depth of 0–0.5m and may have included a dense windblown scum. Samples were shipped overnight on ice and several milliliters (depending on the sample density) were filtered through 0.2 *μ*m Pall Supor 200 and 1.2 *μ*m-pore-size Whatman GF/C 24mm-diameter filters. Filters were stored at –80 °C for later metagenomic sequencing. The culture was established upon sample arrival as described below.

### Culture establishment and maintenance

A culture was established from a 0–0.5m deep bloom sample collected from Anderson Lake on May 20th, 2013. The lake sample was concentrated tenfold by low-speed centrifugation (5,000 RCF). No buoyant cells were observed. Approximately 20 *μ*L of the concentrate was placed on a glass slide. *Anabaena* colonies were individually isolated by serially transferring the aliquot with an automatic pipette between at least five separate 50 *μ*L MilliQ water droplets on the glass slide. Colonies were considered to be isolated when no other cells or cell debris were visible in the surrounding water droplet under 200x magnification on a Zeiss brightfield microscope. Isolated colonies were placed in 200 *μ*L of BG-11_0_ (i.e., BG-11 without nitrogen). BG-11_0_ medium was prepared according to the Susan Golden Lab protocol (UC San Diego). One surviving colony was outgrown in BG-11_0_ for several months, its identity was verified microscopically, and a single colony was again isolated into 200 *μ*L of BG-11_0_. The outgrown colony was then maintained long-term in non-axenic batch culture in BG-11_0_ under white fluorescent illumination of approximately 20 *μEm*^−2^*s*^−1^ at 24 °C with a light/dark cycle of 16hr/8hr. In addition to this culture, Dr. Mike Crayton from Pacific Lutheran University, Tacoma, Washington kindly shared a culture of *Anabaena* AL93 isolated in 1993 on BG-11 agar slants from American Lake, Pierce County, Washington State. It was maintained under the same conditions listed above but in BG-11 medium.

### LC-MS/MS

Filters from lake samples were resuspended by dispersion in 500mL TNE buffer (50mM Tris-HCl (pH 7.5), 100mM NaCl, 0.1mM EDTA). Samples from resuspended filters or cultures were frozen and thawed for three cycles to release intracellular contents. Samples were centrifuged at 5,000 RCF for 5 min, and the supernatant was removed for LC-MS/MS analysis. LC-MS/MS analysis was conducted using a hybrid quadrupole-time of flight instrument (AB Sciex TripleTOF, Foster City, CA) coupled to a Shimadzu Nexera LC-30a UHPLC system (Shimadzu, Columbia, MD). The DuoSpray ion source (AB Sciex, Foster City, CA) was operated in the positive electrospray ionization mode and the following settings were used: ion source gas 1, 40 psi; ion source gas 2, 50 psi; curtain gas, 25 psi; gas temperature, 550 °C; and ion spray voltage, 5500 V. The declustering potential (DP) was 80 V and the collision energy (CE) was set to 27 V. The instrument was operated in positive ion polarity and high-resolution product ion mode. Precursor ion selection was performed in the quadrupole operated at unit resolution. Precursor ions screened included: m/z 166.1 (anatoxin-a, MH ^+^, C_10_H_16_NO ^+^), m/z 168.1 (dihydro-anatoxin-a, MH ^+^, C_10_H_18_NO ^+^), m/z 180.1 (homoanatoxin-a, MH ^+^, C_11_H_18_NO ^+^) and m/z 182.2 (dihydro-homoanatoxin-a, MH ^+^, C_11_H_20_NO ^+^). Product ion mass spectral data were acquired using a scan range of m/z 50–650. Auto calibrations were performed prior to each LC-MS/MS run. Chromatographic separations were carried out using an Agilent Zorbax RRHD SB-18 column (1.8 *μ*m particle size, 2.1x150mm) held at 40 °C. A binary solvent system was used consisting of water (solvent A, Fisher Optima LC/MS grade) and acetonitrile (solvent B, Fisher Optima LC/MS grade), both containing 0.1 % formic acid (98 % pure, Sigma Aldrich). The following gradient was applied: 5 % B hold for 0.5 min then increase to 90 % B within 5 min, reduce to 5 % within 0.5 min and the hold for 5 min. Flow rate was 0.5 mL/min. Sample injection volume was 10 *μ*L.

### DNA extraction and amplification

DNA was extracted from cultures by concentrating the culture tenfold at 40,000 RCF and washing mucilage from the cell pellet with TNE buffer. The cell pellet was resuspended in TNE buffer and treated with a method from Neilan et al. [48] that had the following modifications. The protein fraction was removed with two 25:24:1 phenol/chloroform/isoamyl alcohol extractions followed by two chloroform extractions. Residual phenol was removed with a final diethyl-ether extraction. Total DNA from lake samples used for metagenome analysis was extracted from 1.2 *μ*m-pore-size filters by macerating the filters with a pestle and extracting DNA as described.

### DNA sequencing

Samples are listed (Table 1). Each Illumina library was prepared and sequenced at the Oregon State University Center for Gene Research and Biotechnology, Corvallis, Oregon. The *Anabaena* sp. WA102 culture was also sequenced using the PacBio C6-P4 long-read sequencing platform at the Washington State University Molecular Biology and Genomics Core, Pullman, Washington. Prior to PacBio sequencing, DNA fragments were size-selected on the BluePippin system (Sage Science) to enrich for reads longer than 8 kbp. Raw reads were collected from four PacBio SMRT cells.

### Draft genome binning

Illumina metagenomes were assembled using idba version 1.1.1 assembler software [49] on a 64-bit Linux server with 500GB of RAM. Prior to assembly, any reads containing ambiguous basecalls ("N") were culled. The large chromosome from the *Anabaena* sp. 90 genome was used as a reference to guide assembly. Within idba, assemblies with kmer sizes ranging from 20nt to the sequence read length (100nt to 250nt) in 10nt increments were combined in the final assembly. Sequencing data from four PacBio SMRT cells for the *Anabaena* sp. WA102 culture was self-corrected, assembled, and polished using the Hierarchical Genome Assembly Process (HGAP) Pipeline at the Washington State University Molecular Biology and Genomics Core. Reads from original fastq files were mapped to the Illumina and PacBio assemblies using bwa version 0.7.5a-r405 [50]. Average coverage depth for each contig was calculated using samtools version 0.1.18 (r982:295) and the calc.coverage.in.bam.depth.pl script from the mmgenome package (https://github.com/MadsAlbertsen/mmgenome) [51]. The mmgenome network.pl script generated a network of contigs based upon paired-end read data extracted from the bwa-generated SAM file. Bacterial and archaeal metagenome contigs were taxonomically classified using the PhylopythiaS+ support vector machine (SVM) classification software with only a contig fasta file and not a scaffold fasta file (https://github.com/algbioi/ppsp) [52]. 16S marker genes were detected in the contig file and used by PhylopythiaS+ to select an SVM training dataset automatically. Putative protein coding sequences were identified in each assembly fasta file using Prodigal version 2.6.2. To identify essential genes, putative protein sequences were aligned against a curated hmm database from the mmgenome package with the HMMER version 3.0 package (http://hmmer.janelia.org/) [53]. A custom data generation shell script based on the data.generation.2.1.0.sh script from mmgenome was used to combine the above processes (https://github.com/russianconcussion/data.analysis.scripts/blob/master/mmgenome.datagen.sh). Average coverage depth, network, taxonomic classification, and essential gene data for each assembly were imported into a data.frame structure in R. Finally, the mmgenome R package was used to generate a plot of genome clusters within the metagenomes, define and evaluate completeness of the clusters, and export well defined genome clusters as contigs in fasta format. Genome clusters in fasta format were annotated using Prokka version 1.11 [54].

### Finished *Anabaena* sp. WA102 genome analysis

The finished *Anabaena* sp. WA102 genome was annotated using Prokka version 1.11 and the NCBI Prokaryotic Genome Annotation Pipeline after submission to Genbank. Non-ribosomal and polyketide synthesis gene clusters were annotated using the AntiSMASH webserver (http://antismash.secondarymetabolites.org/) [55]. The genome was scanned for prophages and genomic islands using the PHAST (http://phast.wishartlab.com/) and IslandViewer 3 (http://www.pathogenomics.sfu.ca/islandviewer/) webservers [56, 57]. Insertion sequences were manually annotated with the IS Finder database [58]. BLASTN and CIRCOS were used to detect local alignments between *Anabaena* sp. WA102 and *Anabaena* sp. 90 and plot the corresponding similarities (http://circos.ca/) [59, 60]. BLASTN, GenomicRanges, and CIRCOS were used to detect large repeat regions within the *Anabaena* sp. WA102 genome and map the *Anabaena* sp. WA102 Illumina assembly contigs to the finished genome [61]. Long and short repeat regions were also detected using RepeatScout to model repeat regions and RepeatMasker to annotate them (http://www.repeatmasker.org) [62]. Protein domains within the AnaG protein were identified with the SMART online protein domain database [63]. Whole genomes were aligned using Mauve 2.4.0 on default settings and Gepard 1.30.

### Comparative genomics among members of the *Nostocaceae*

The putative protein-coding contents of *Anabaena* sp. WA102, *Anabaena* sp. AL93, *Dolichospermum* sp. AWQC131C, and *Dolichospermum* sp. AWQC310F was annotated using Prokka version 1.11. Protein content from *Anabaena variabilis* ATCC 29413, *Anabaena* sp. 90, *Anabaena* sp. PCC 7108, *Anabaena cylindrica* PCC 7122, *Nostoc* sp. PCC 7107, *Nostoc* sp. PCC 7120, and *Nostoc* sp. PCC 7524 were downloaded from Genbank. Protein-coding contents from each of the eleven genomes were used to build a phylogenomic tree. The bi-directional best BLASTP-hits method was used to identify orthologs in each genome [64]. These were clustered with the MCL algorithm and aligned with muscle [65, 66]. MCL inflation parameters were used as in the HAL paper ([67]; there were 13 inflation parameters), groups were selected starting from the highest moving to the lowest inflation parameter, selecting groups containg bidirectional best hits and no paralogs. BLAST settings were as found in [68], with an e-value threshold of 0.1. Protein alignments were masked with zorro to reduce noise from uninformative amino acid alignment positions and checked for a best fit among protein evolution models with ProtTest version 3.1 [69, 70]. The best-fit protein evolution model was used in RAxML to generate the final tree, which was rooted within the *Nostoc* genus outgroup at *Nostoc* sp. 7107, in accordance with Shih et al. [10, 71]. Proteins were also mapped to the free KEGG database from 2011 and compared across metabolic pathways [72]. A grid that correlates highlighted KEGG comparisons with the phylogenomic tree described above was generated using the adephylo package in R [73].

### Accession numbers used in study

*Anabaena* sp. WA102 [Genbank:CP011456-7], *Anabaena* sp. AL93 [Genbank:LJOU00000000], *Dolichospermum* sp. AWQC131C, *Dolichospermum* sp. AWQC310F, *Anabaena variabilis* ATCC 29413 [Genbank:NC007413], *Anabaena* sp. 90 [Genbank:NC_019427 and Genbank:CP003285], *Anabaena* sp. PCC 7108 [Genbank:KB235895], *Anabaena cylindrica* PCC 7122 [Gen-bank:NC_019771], *Nostoc* sp. PCC 7107 [Genbank:NC_019676], *Nostoc* sp. PCC 7120 [Genbank:NC_003272], *Nostoc* sp. PCC 7524 [Genbank:NC_019684], *Anabaena* sp. 37 anatoxin-a region [Genbank:JF803645], *Oscillatoria* sp. PCC 6506 anatoxin-a region [Genbank:FJ477836], *Cylindrospermum* sp. PCC 7417 [Genbank:NC_019757], and WA25 metagenome sample [SRA:SRP066506].

## Abbreviations

NRPS, Nonribosomal peptide synthase; PKS, Polyketide synthase; SMRT sequencing, Single-molecule real-time sequencing; ANI, Average nucleotide identity; LCB, Local colinear block

## Additional file

Additional file 1 Additional Figures 1-8 provide supporting information for the manuscript Brown et al., 2016, Structural and functional analysis of the finished genome of the recently isolated toxic Anabaena sp. WA102. (PDF 26100 kb)
