# Supplementary material for: Structural and functional analysis of the finished genome of the recently isolated toxic Anabaena sp. WA102
Source: BMC Genomics. 2016 Jun 13;17:457. doi: 10.1186/s12864-016-2738-7 (PMC4907049; doi:10.1186/s12864-016-2738-7)
Supplement: Additional file 1 — Additional Figures 1-8 provide supporting information for the manuscript Brown et al., 2016, Structural and functional analysis of the finished genome of the recently isolated toxic Anabaena sp. WA102. (PDF 26100 kb) [file 12864_2016_2738_MOESM1_ESM.pdf]

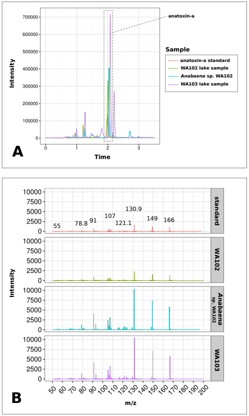

Figure 1: HPLC-MS/MS survey of anatoxin-a and derivatives. **A)** HPLC elution of compounds extracted from the *Anabaena* sp. WA102 culture and two Anderson Lake samples (WA102 and WA103). Anatoxin-a elutes at approximately 2 minutes, as indicated by the anatoxin-a standard. Anatoxin-a peaks are surrounded by a gray dashed line. No variants of anatoxin-a were detected. **B)** Ion mass spectra for anatoxin-a are compared from lake sample WA102 (May 20th, 2013 with 12.5 μg/L anatoxin-a), lake sample WA103 (June 17th, 2013 with 35.8 μg/L anatoxin-a), and the culture. All spectra match the spectrum of the anatoxin-a standard closely.

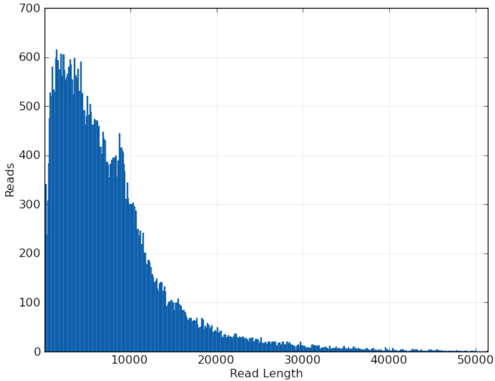

Figure 2: PacBio read length distribution for the *Anabaena* sp. WA102 culture. PacBio read length average 8.5 kbp, allowing complete assembly of the *Anabaena* sp. WA102 across long repeat regions.

Chromosome

Cluster	Genome coordinates	Type	Putative product
1	25089-69723	Other	Exopolysaccharide for sheath
2	518674-570979	Type I PKS	Heterocyst glycolipid
3	878154-925068	Ketide synthase	Heterocyst glycolipid
4	994957-1015889	Terpene synthase	Unknown
5	2357970-2406282	NRPS	Predicted same as 14
6	2792407-2820462	Other	Cyanobactin
7	2976022-2997929	Terpene synthase	Unknown
8	3134077-3154907	Terpene synthase	Unknown
9	3214154-3268375	NRPS	Unknown
10	3903629-3950105	NRPS	Unknown
11	4351144-4407332	Type I PKS	Anatoxin-a
12	4990274-5031965	Other	Unknown
13	5199666-5210010	Bacteriocin	Unknown
14	5529600-5576031	NRPS	Predicted same as 5

Plasmid

Cluster	Genome coordinates	Type	Putative product
1	1-51077	NRPS	Unknown

Table 1: Secondary metabolite synthesis gene clusters identified in *Anabaena* sp. WA102 by antiSMASH v3.0.0.

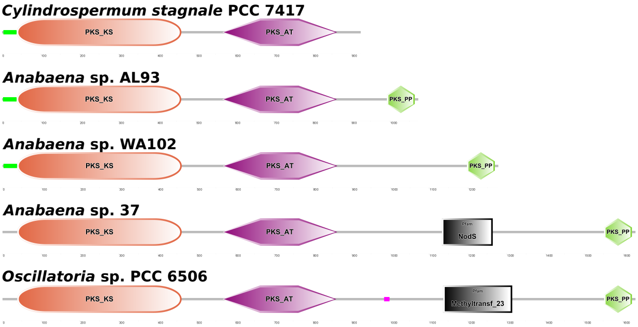

Figure 3: Comparison of the AnaG protein domains among Cyanobacteria. The AnaG protein sequences from *Oscillatoria* sp. PCC 6506 and *Anabaena* sp. 37 have methyltransferase domains not present in any other AnaG protein sequences. The methyltransferase domains are divergent. The methyltransferase in *Oscillatoria* sp. PCC 6506 is proposed to contribute a methyl group that makes the homoanatoxin-a variant of anatoxin-a. AnaG lacking a methyltransferase domain (or containing a non-functional domain) likely prevents production of homoanatoxin-a. In support of that, no homoanatoxin-a was detected in the *Anabaena* sp. WA102 culture.

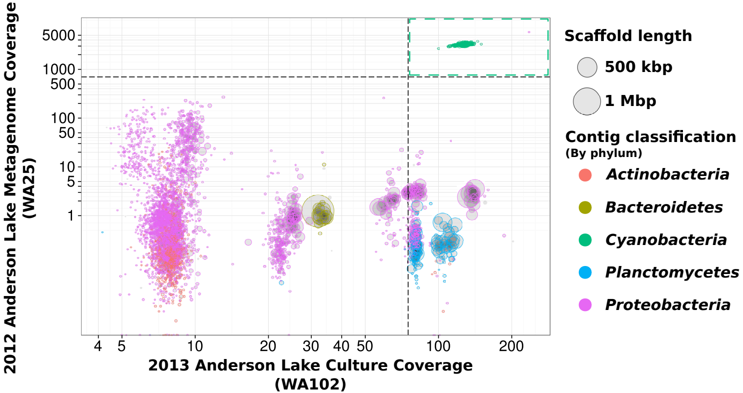

Figure 4: Contigs assembled from Illumina short reads of the *Anabaena* sp. WA102 culture metagenome. Average coverage of contigs by reads from the WA25 metagenome is plotted along the ordinate. Average coverage of contigs by reads from the *Anabaena* sp. WA102 culture metagenome is plotted along the abcsissa. Contigs from the *Anabaena* sp. WA102 genome can be binned from the metagenome by selecting contigs with greater than 70x coverage from the *Anabaena* sp. WA102 culture metagenome (black vertical dashed line) and greater than 700x average coverage from the WA25 metagenome (black horizontal dashed line). The genome bin is boxed by the green dashed line. Summary statistics of the genome bin are shown in Table 1.

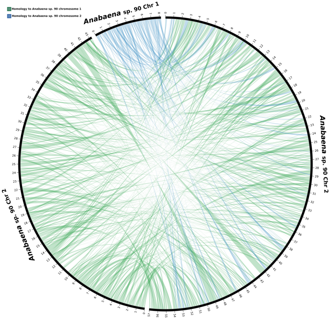

Figure 5: Nucleotide alignment between *Anabaena* sp. 90 and WA102. Although *Anabaena* sp. 90 and WA102 share 91.5% average nucleotide identity, they nearly entirely lack synteny. Additionally, the *Anabaena* sp. 90 genome is divided between two chromosomes, unlike the single chromosome of the *Anabaena* sp. WA102 genome.

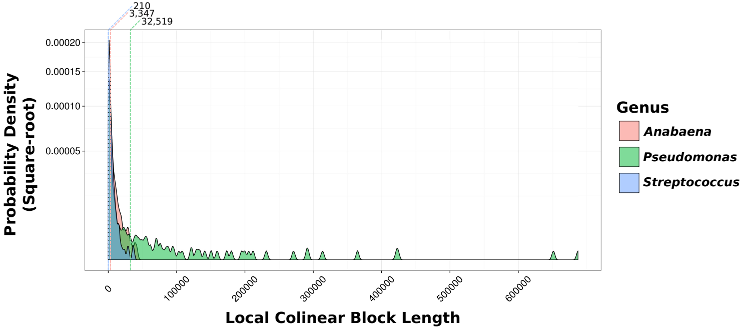

Figure 6: Probability density of the local colinear block (LCB) lengths for three bacterial genera. The same pairwise genomes comparisons from the dotplots in Figure 9 are aligned in Mauve. Mauve generates LCBs, which are syntenous regions defined by conserved termini, and that may contain large insertions. The lengths of these LCBs are plotted in a probability density plot for each pairwise genome comparison. The mean LCB length for each pairwise genome comparison is shown as a dotted line with the value printed above the graph. *Pseudomonas* genomes have a mean LCB length of 32.5 kbp, *Anabaena* 3.3 kbp, and *Streptococcus* 210 bp, quantifying what can be observed in the dotplots.

LCB size	*Anabaena* sp. WA102 coordinates	*Anabaena* sp. 90 coordinates
24724	1179150-1203874	2682853-2688083
24367	2614593-2638960	556126-567297
19543	747882-767425	1249180-1268773
17895	2513551-2531446	1869250-1880948
15774	4233850-4249624	3964808-3976035
14557	1992912-2007469	3575881-3591878
14037	128638-142675	2737445-2752527
13886	2997345-3011231	1702722-1715778
13370	1572453-1585823	1408076-1418961
13344	804588-817932	217041-228577

Table 2: The nucleotide coordinates of the ten longest local colinear blocks (LCBs) calculated by Mauve between *Anabaena* sp. WA102 and *Anabaena* sp. 90. The longest LCB includes a cryptic prophage unique to *Anabaena* sp. WA102.

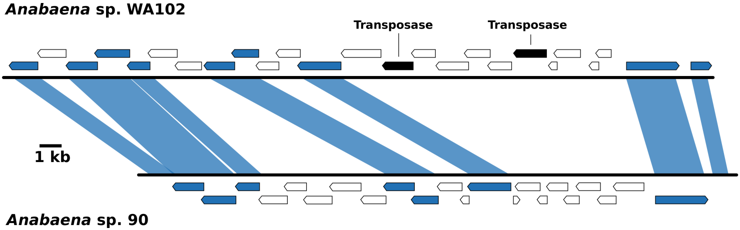

Figure 7: Comparing synteny within a local colinear block between *Anabaena* sp. WA102 and 90 (nucleotides 1,179,150-1,203,874 and 2,682,853-2,688,083, respectively). Within this local colinear block, there is evidence of interruption by transposases. Most of the six instances of broken synteny in this LCB are not clearly attributable to a particular mechanism.

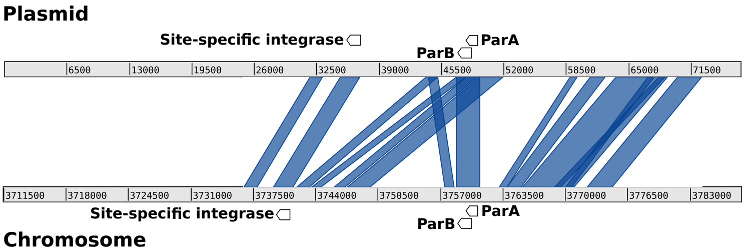

Figure 8: Nucleotide alignment between *Anabaena* sp. WA102 chromosome and plasmid. Nucleotide similarity between the chromosome and the plasmid indicates that the plasmid may be integrative and form genomic islands either by integrating into a site on the chromosome or by homologous recombination with the chromosome. The plasmid may be integrative because it encodes site-specific integrases, which can also be found at the homologous site on the chromosome.
